# Parents’ Experiences of Change in Developmental and Transactional Processes After Time-Limited Intersubjective Child Psychotherapy – A Qualitative Study

**DOI:** 10.3389/fpsyg.2022.898389

**Published:** 2022-07-13

**Authors:** Charlotte Fiskum, Unni Tanum Johns, Tonje Grønning Andersen, Karl Jacobsen

**Affiliations:** ^1^Department of Psychology, Norwegian University of Science and Technology, Trondheim, Norway; ^2^Child and Adolescent Mental Health Services, Akershus University Hospital, Oslo, Norway; ^3^Department of Psychology, University of Oslo, Oslo, Norway

**Keywords:** psychodynamic therapy, parental work, play therapy, affect integration, regulation, mentalization

## Abstract

Psychopathology in children cannot be understood without considering developmental processes and transactional relationships, particularly the relationship with caregivers. Time-limited intersubjective child psychotherapy (TIC) is a developmental and transactional approach aimed at helping children and caregivers get back on healthier developmental trajectories. Core developmental processes, such as self-other-regulation and affect integration, are considered particularly important for healthy function and transactions with caregivers and contexts. Therefore, TIC seeks to strengthen core developmental processes in the child and the caregivers’ ability to scaffold the child’s development. This is achieved through parallel child therapy and parent sessions. The current study is a qualitative study of parents’ experiences of change after TIC. The study explores parents’ perception of change in their child, themselves, and their transactions, and what they experienced as helpful in therapy.

**Materials and Methods:**

Nine semi-structured qualitative interviews were conducted with parents (*n* = 13) of nine children aged 9–12 years with internalizing difficulties after completing TIC. The data were analyzed with thematic analysis.

**Results:**

The parents described positive changes in the children’s ability to understand, regulate and express themselves. The parents also described positive changes in their parenting, and for all nine children the parents reported positive changes in the transactions between themselves and the child. Most parents saw the parallel child and parental sessions as particularly important, while several parents mentioned play and the therapeutic focus.

**Discussion:**

Parents’ description of changes after TIC indicate that the parents perceived their children as strengthened in core developmental processes important for intersubjective exchanges such as self-regulation and affect integration. In addition, parents saw their children and their own contributions more clearly, and their transactions were described as more supportive and positive.

**Conclusion:**

The results from this study give support to TIC as a developmentally supportive approach to therapy, with potential effects on children’s core developmental processes, their parents’ ability to both see and scaffold the child’s development and positive effects on the transactions between children and parents. The positive effects likely result from the integration of the different parts of TIC and the synergies taking place between them, rather than any one component by itself.

## Introduction

The current study investigates parental perceptions of change after time-limited intersubjective child psychotherapy (TIC). Parents’ perception of change is important because psychopathology in children can be understood as disturbances in core developmental processes that can both affect and arise from transactions between a child and caregivers and their context over time (Sameroff, 2009). Therefore, symptoms or disorders must be seen and understood as part of a transactional system – never residing solely in either child or environment. Instead, psychopathology is rooted in and continually unfolds from the transactions between child and context. In children and adolescents referred to therapy, core developmental processes such as adequate regulation of attention, arousal, and affect can often present as disturbed, derailed, or halted (Schore and Schore, 2008; Jacobsen and Svendsen, 2010). The same is often true for their important social relationships. Therefore, therapy with children should bring developmental processes back on track and ensure developmentally supportive transactions between the child and their social context. A developmental view of psychopathology also means that therapy with children and young people should be viewed as a *developmental process* rather than a repair process with a one-sided focus on symptom relief (Johns and Svendsen, 2016). Specifically, this view of psychotherapy includes a focus on influencing core developmental abilities and processes underlying developmentally supportive transactions (Jacobsen and Svendsen, 2010), such as the capacity for joint attention (Mundy and Jarrold, 2010; Hansen, 2012), implicit beliefs around oneself as a causal agent (self-agency) (Stern, 1985/2000; Kögler, 2012), and regulation of arousal and affect (Sørensen, 2006; Schore and Schore, 2008). Affect integration (Solbakken et al., 2011a), defined as the ability to attend to, tolerate, regulate, and express affect both nonverbally and conceptually, is particularly important because of the central role of affect in both communication and creation of meaning and significance around internal and external events (Saarni et al., 1998; Campos et al., 2004).

Furthermore, as parents are a large part of a child’s context and day-to-day transactions, parents’ ability to see, meet, and reflect on the child’s developmental needs is vital for healthy development (Sørensen, 2006; Schore and Schore, 2008). Importantly, transactions and transactional effects can be positive or negative (Sameroff, 2009). When families experience difficulties around a child, negative transactions, such as accusations, arguments, misunderstandings, or escalations of relational problems, are common. These negative transactions can, in turn, trigger vicious cycles with adverse effects on the child’s natural development. For instance, parents can lose faith in the child’s developmental possibilities or lose confidence in their parenting abilities or ability to interact as a family. Negative transactions can also lead to the parents dreading interactions with the child or trying to pre-emptively control or avoid interacting with the child in general or in specific situations. The child may also lose faith in itself, the parents, or the future and try to gain control of the situation, which may further exacerbate difficulties (e.g., when both parties “turn up the volume” no one is heard). Importantly, even when a child experiences improvement in therapy, negative transactions may persist if parents are not included in the therapeutic process, as parents may still see and meet the child and the situation as it was. Therefore, parental involvement in child psychotherapy is essential (Slade, 2008; Hafstad and Øvreeide, 2011; Johns and Svendsen, 2016).

Time-limited intersubjective child psychotherapy (TIC) is a therapy approach based on a developmental, transactional, and intersubjective understanding of the therapeutic process (Hansen, 2012; Svendsen et al., 2012; Johns and Svendsen, 2016). The focus in TIC is on stimulating and supporting developmentally important processes and transactions that, for one reason or another, have become disturbed, helping the child and parents return to a healthier developmental track (Svendsen et al., 2012; Johns and Svendsen, 2016). In this understanding, therapy represents a scaffolding and integrational learning experience that allows children and parents to experience growth together, both in and outside of therapy (Hansen, 2012; Johns and Svendsen, 2016).

*Intersubjectivity* in TIC refers to the sharing of subjective experiences. Bodily nonverbal expressions and affect are particularly important in understanding intersubjectivity in TIC due to their primary role in developing a sense of self, in relationships, and in co-regulation (Stern, 1985/2000; Schore and Schore, 2008). Furthermore, sharing attention, intentions, and affective states is central to intersubjective transactions. Resulting from decades of experiential and theoretical work in clinical child psychology in Norway, TIC served as an inspiration in the development of time-limited mentalization-based therapy for children (Midgley et al., 2017). A differentiating factor between the two approaches is the explicit and pervasive focus on core developmental processes assumed to precede and support the development of mentalization and healthy transactions in TIC, such as self-agency, non-verbal intersubjective relating, sharing of attention, and co-regulation of arousal and affect through nonverbal communication.

In TIC, parallel sessions with children and parents promote awareness in both the child and parents of implicit subjective processes and experiences underlying patterns in current intersubjective relating (Johns and Svendsen, 2016). The aim is to increase the parent’s capacity to relate intersubjectively with the child, for example by following the child’s attention and taking time to feel what the child is feeling. Concrete episodes occurring during the course of therapy are used to help the parents reflect on the child’s experiential and subjective world and expressivity. At the same time, TIC considers that the child’s perspective differs from that of adults and facilitates child-centered therapy sessions following the child’s initiative and activity. Whereas the parental work emphasizes reciprocal dialogue and mentalizing capacity, therapy sessions with the child include nonverbal play and communication emerging from the child’s unique perspective. Adjusting to micro-processes in the here and now in sessions provides new intersubjective experiences for the child that may change previous implicit negative relational patterns or expectations of negative transactions or failures in intersubjectivity (Johns, 2018).

As a therapy approach, TIC has three distinct characteristics: Time- limitation, a clear and agreed-upon focus for therapy, and parallel parent-work, described in depth in the Norwegian handbook (Johns and Svendsen, 2016) and presented below. The first main characteristic, *time-limitation*, refers to having a predictable and explicit time frame for the duration of therapy. The time-limitation aims to give the child and parents a clear overview of sessions, providing a frame around the therapy while also making the work directed. A therapy calendar is provided, where the child can make drawings or write something from each session. The actual time frame may vary, but 12 h of therapy is usually recommended (Johns and Svendsen, 2016). These 12 sessions are divided into an initial phase, a middle phase, and an ending phase. Children in need of more than 12 sessions can be given more than one course of TIC.

The second main characteristic of TIC is that therapy must always have a clear and agreed-upon *therapy focus*. The focus is established based on the child’s experiences and expressions, information from the parents, and the therapist’s observations from the clinical-psychological interview (see below). The focus is formulated as a metaphor based on the child’s language and expressivity and aims to increase the child’s engagement in and understanding and ownership of the therapy process (Johns and Svendsen, 2016). The focus should provide direction for the therapy process and be understandable and meaningful for the child and parents, and is an essential part of building a solid working alliance (Bordin, 1979) with the child and with the parents and family as a whole. Typically, different therapeutic foci are centered around the development of (a) self-agency or self-regulation, (b) emotional areas or affects which the child is not able to be in contact with or express, or (c) helping the child gain a sense of coherence and meaning in their self-narrative. The focus is resource-focused, not problem-focused, and developmentally oriented. One example of a therapy focus aimed at increasing self-agency and emotion regulation was formulated as “becoming just strong enough.” It was inspired by a child’s comments about feeling weak. Notably, the focus should never be formulated in a way that is hard to work with in therapy or might easily fail (for instance, “I should make more friends” or “I should do better in school”). Finally, it is vital that the child sees the parents accept the focus and that they give the child explicit permission to work on the focus.

The third main characteristic of TIC is *parallel parental work*. It means that both child and parents are given their own physical and mental space where experiences can be shared, validated, and reflected upon. Parents meet their own parent-therapist in parallel with the child’s therapy sessions. The parent sessions aim to support healthy parent behaviors and search for and create possibilities for expanding the parents’ reflexive functioning and intersubjective sensitivity. The parents’ ability to observe and regulate their affect, especially their negative affect, is important for whether the interaction will be experienced as emotionally supportive and regulating or not (Schore and Schore, 2008). How the parent perceives the child, its intentions, and behaviors, as well as their own, i.e., the parents’ mentalizing capacity (Fonagy et al., 2002; Midgley et al., 2017), also affects the transactions positively or negatively. The number of parallel sessions with parents is more flexible than for the child. However, a minimum of five sessions is recommended. The parent sessions help thematize the child’s developmental history and the parents’ understanding of the child’s challenges, resources, and developmental needs. Parents need to feel safe and validated to support development through exploration and sharing. Therefore, while the parent sessions are centered on the child, it is important that the parents themselves also experience being understood and met and that they are helped to express their feelings toward the child and reflect on the child and their relationship. To reflect together with parents and try to arouse curiosity about the inner experiential world of the child is a way to move beyond behavior. The main aim is to help the parents get back on track of the child’s developmental opportunities and strengthen their ability to recognize, provide and engage in developmentally supportive transactions with the child (Johns and Svendsen, 2016).

Before therapy starts, there are three initial *clinical-psychological interview sessions* with the child and parents separately. The clinical-psychological interview with the child is centered around the child’s subjective experiential world, interests, concerns, and thoughts around the present, past, and future as well as the coming therapy. It is called an interview to recognize the child as an active participant in exchanges concerning the child’s life. Importantly, these sessions are not a structured or formal interview-situation but sessions aimed at getting to know the child and their intersubjective and transactional/relational capacity through play and varied interactions. Since children express themselves mainly implicitly and nonverbally, there is an emphasis on facilitating individual communicative possibilities. During the clinical-psychological interview, the therapist focuses on building trust and a basis for a therapeutic relationship through validating the child’s expressions. The therapist is also focused on understanding the child’s developmental needs. This is achieved through the therapist’s explicit focus on core developmental processes in the child, such as the ability to direct attention, establish joint attention, and express emotions nonverbally through play and verbal capacity. Also, the child’s apparent affective range, awareness, tolerance, and expressivity and the child’s ability to regulate affect and arousal are assessed.

The initial interviews with parents aim to illuminate the child’s developmental history and potentially negative aspects and contextual factors related to the present circumstances underlying the referral to psychotherapy. Also, thoughts and feelings about the child and the family situation are clarified. Here the parents’ understanding of the child’s developmental history and needs, past and current challenges and resources, and the relational and transactional history is in focus. It is generally recommended to have two therapists per patient during the whole course of therapy when working with TIC, one for the child, and one for the parents, starting with the initial interviews. As the child therapist is conducting the clinical-psychological interview with the child, the designated parent-therapist meets with the parents. One important task for both therapists is to collaborate in order to bridge between parent and child therapies.

*The current study, aims, and research questions:* Because of the importance of developmental core processes and transactions in child psychopathology and parents’ essential role in seeing and supporting the child’s development, the current study explores parents’ experience of change after TIC. In particular, the focus is on the parents’ descriptions and reflections around changes in the child, themselves, and changes in child-parent transactions outside of therapy. In addition, the study investigates what parents perceived as helpful or unhelpful aspects of therapy. The study is based on a thematic analysis of nine interviews with 13 parents whose children received TIC for internalizing difficulties. The main research questions are informed by an understanding of TIC as a developmental and transactional approach to child psychotherapy aimed at generating both individual change in children (particularly in developmentally important processes) and their parents, as well as changes in their transactions, and are:

(*1*) *What changes, if any, do parents describe in their children after TIC?*
(*2*) *What changes, if any, do parents describe in themselves after TIC?*
(*3*) *What changes, if any, do parents describe in transactions between themselves and their child after TIC?*
(*4*) *What do parents describe as helpful or not in TIC?*
(*5*) *Do the parents mention other salient themes of relevance?*

## Materials and Methods

### Ethics

The regional ethics board for medical and health-related research (REK, region North) approved the study. The study complied with the Helsinki declaration for research ethics (World Medical Association, 2013). All parents were given an oral and written description of the project and gave informed consent to participate.

### Design and Subjects

Parents whose children were enrolled in the “Child Psychotherapy Project at NTNU” (Fiskum et al., 2017, 2021) were invited to participate in interviews before and after therapy. The children enrolled in the main project were between 9 and 13 years of age and deemed to suffer from internalizing difficulties based on parent reports on the child behavior checklist (CBCL) (Achenbach and Rescorla, 2001) (scores elevated to borderline clinical and clinical levels) and descriptions of internalizing difficulties as evaluated by specialists in clinical child psychology.

The recruitment to participate in interviews took place at the start of the project. Parents of sixteen children were asked to participate in the voluntary interviews. Parents of nine of the children agreed to attend interviews after therapy, while seven families declined. An inspection of therapist-reported change did not indicate differences in therapeutic efficacy between the families that participated and those that did not, indicating no systematic bias in participation related to the effect of therapy. In the non-participating group, 7 out of 7 children were described as showing positive change and in no further need of more therapy. In the participating group, 8 of 9 children were described with positive changes after therapy, and eight were assessed as not needing further therapy.

Four interviews were conducted with both mothers and fathers present, four interviews were conducted with only mothers, and one interview was conducted with only a father, meaning that a total of 13 parents (eight mothers and five fathers) from nine families were interviewed in nine separate interviews. All the families were from the Norwegian majority culture. Five of the mothers had completed education at master’s degree level, one at bachelors’ level, and two at high school/vocational school level. Three fathers had completed master’s degree education, and two had high school/vocational school. Two of the families had divorced parents. After the three clinical-psychological interview sessions, the children received 10 weekly individual therapy sessions, the parents received between 2 and 12 parallel parent sessions (averaging at 5.7 sessions), and each family received 2–3 joint sessions including the child, parents, and both therapists. Table 1 presents case characteristics for each child using fictitious names, along with the therapist’s evaluations at the end of therapy.

**TABLE 1:** Case characteristics.

Case and age	Described difficulties at referral and previous help received	Therapists’ evaluation at the conclusion of therapy
*Ella*, 9	Ella was referred due to sadness/mild depression and separation anxiety. She was frequently angry. The parents were divorced and experienced difficulties in their co-parenting. Ella had received some follow up from a school nurse before the referral. Her therapy-focus was “*we will work together exploring all the powers of Ella’s heart”*	Ella was described as more regulated at the end of therapy. Her parents were described as better able to see her and meet her needs. They also seemed to co-operate better. She was not seen as in need of further therapy

*John*, 9	John was referred with anxiety. He was often very agitated and had problems calming himself. His anxiety had led to him not eating and becoming very thin. He had not received any mental health help previously. His therapy-focus was *“we will explore what is it like to be 9 and have experienced everything John has experienced”*	John started eating again during therapy and was seen as showing less anxiety and better regulation overall. Still, the parents and therapist agreed that he should continue in therapy to consolidate the changes further

*Monica*, 9	Monica was referred with somatic symptoms that were deemed stress-related and bouts of sudden sadness and crying that were hard to regulate. There was a suspicion of possible trauma, but this suspicion was significantly weakened during the assessment and therapy. Her parents were divorced but reported co-parenting well. Monica had been assessed at the local somatic ward for her somatic symptoms and had received some follow up from a school nurse before therapy. Her therapy-focus was “*we will work together to find all your inner treasures”*	At the end of therapy Monica’s somatic symptoms had resolved and she was described with less dysregulated emotion and crying. She was not seen as in need of further therapy and there was no further suspicion of trauma

*Matt*, 10	Matt was referred with anxiety and sadness and outbursts of strong negative emotions. He was frequently in conflict with other students and avoiding school. He had not received any mental health help previously. His therapy-focus was *“we will work together so you can take more control of the war within you, making it more peaceful”*	At the end of therapy Matt was seen as less anxious and more vital and regulated and he functioned better in school. He was not seen as in need of further therapy

*Anne*, 10	Anne was referred with generalized anxiety and several phobias. She showed frequent outbursts of anger. Anne had received some follow up from a school nurse before the referral. Her therapy-focus was *“we will work together to find the right pace for Anne*”	Anne was described as more regulated at the end of therapy, and her parents were described as better able to support and regulate her. She was not seen as in need of further therapy

*Kathryn*, 10	Kathryn was referred with separation anxiety and difficulties calming herself at night causing sleep problems, sadness, and somatic symptoms. She had not received mental health help before. Her therapy-focus was “*we will get to know both the little and the big Kathryn*”	At the end of therapy, the therapist described Kathryn as still anxious, but better able to express herself. Her somatic symptoms had subsided. The parents were described as better able to support Kathryn. She was not seen as in need of further therapy

*Siri*, 11	Siri was referred with a specific phobia and a suspicion of generalized anxiety. She had previously received exposure therapy but was still symptomatic at referral. The therapeutic assessment was inconclusive as to the nature of her anxiety. Her therapy-focus was “*we will explore all of Siri’s rooms together”*	It was not clear to what degree Siri experienced a relief in anxiety after therapy, but Siri’s parents were described as better able to help Siri seek out and tolerate anxiety-provoking situations at the end of therapy. She was not described as in need of further therapy, instead focusing on the importance of continued parental support

*Vicky*, 11	Vicky was referred with anxiety, somatic symptoms, sleep problems, difficulties regulating her arousal and frequent bouts of anger that could get physical toward her parents. She was also described as disorganized, chaotic, and not very assertive. There was a history of mental illness in the close family and Vicky had received 10 group sessions for children as next-of-kin before therapy. Her therapy-focus was *“we will work on putting on the visibility cape together*”	Vicky was described as calmer and less chaotic at the end of therapy. She was described as better able to describe her feelings and her somatic symptoms had subsided. Her parents were described as better able to support her. She was not seen as in need of further therapy

*Sean*, 12	Sean was referred with mixed symptoms of anxiety and depression, including high physical arousal, difficulties sleeping, nausea, and frequent crying. Sean had received some follow up from a school nurse before therapy. His therapy-focus was *“we will work together to get to know all the different notes in your song”*	At the end of therapy Sean was seen as better able to tolerate and understand his emotions leading to decreases in anxiety, sadness, and somatic symptoms. His parents were described as supportive in his development. He was not seen as in need of further therapy

*All names are fictitious, and identifying details were not included.*

### Interviews

The interviews were semi-structured and investigated the parents’ experiences of TIC, their perception of changes after TIC, and what they thought contributed to the changes. The interviews also asked about parents’ expectations to and perceptions of child psychotherapy in general. The interviews lasted between 15 mins and up to 1 h, with 30 mins per interview average running time. The interviews were conducted in Norwegian by an experienced clinical child psychologist (female). The interviewer was not involved as a therapist in the project. The interviews were transcribed in full, yielding transcripts between 9 and 20 pages long. The parents’ expectations regarding psychotherapy were also explored in short pre-therapy sessions focused on parental expectations and practical information. Here both parents attended all but one interview, and a total of nine mothers and eight fathers were interviewed. The parents were also given practical information on the upcoming therapy schedule in these sessions. The length related to expectations was not recorded but is estimated at between 5 and 15 mins per interview. An overview of the parents’ expectations before therapy is presented first in the “Results” section to provide more context around the results.

### Analysis of the Data From the Interviews

The analysis was done using thematic analysis (Braun and Clarke, 2012) by the first author, a clinical child psychologist with experience with TIC, but no involvement as a therapist in the project. The interviews were analyzed in Norwegian and then translated into English for inclusion of excerpts in the article. Names and identifying details in the interviews were changed to secure anonymity. The analysis followed a 5-step process with familiarization with the interviews through reading and rereading every interview, followed by emergent, data-driven coding of the material in Nvivo (2020). The coding consisted of generating, defining, and naming themes before reviewing and condensing these themes into overarching themes. The analysis was inspired by a stepwise approach (Tjora, 2018), and codes were generated close to the data by bracketing theoretical assumptions in the first stages of analysis. In later stages of the analysis the apparent validity of the extracted themes was examined considering theory on the importance of intersubjectivity, self-other-regulation, and transactions for development. The theoretical foundation was used as “*a broad orienting framework for exploring topical areas without imposing preconceived ideas and biases*” (Wu et al., 2016). An illustration of the analytic process can be seen in Table 2. The analysis focused on each research question in turn. Finally, the remaining authors read, discussed, and approved the analysis.

**TABLE 2:** Illustration of the analytic process.

Early stages: Reading and approaching the data with theoretical assumptions bracketed aiming for inductive closeness to the data	Identifying raw themes and units of meaning	Condensation of meaning	Subtheme	Main/Overarching theme	Later stages: Approaching the coding and themes with awareness of theory aiming for more abductive reasoning
		
	*Parent: “It has been a lot - she is not SO… that tension in the body, in a way”* *I: Yes.* *Parent: “It - she’s calmer”*	Describes child as calmer and less tense	Improved regulation of arousal and behavior	Perceived changes in the regulation of arousal and behavior	
		
	*Parent: “And less… like before, she could be very, like, when she had her outbursts that she could approach us physically, I was going to say. That - not that she attacked me-”* *I: No -* *Parent: “Simply that she came so close that she walked into me or was a bit like that - And she has not done that at all anymore”*	Describes less aggressive outbursts	Less aggression and outbursts	Perceived changes in the regulation of arousal and behavior	

*I, interviewer.*

## Results/Themes

The identified themes and subthemes are presented according to the research questions, along with non-exhaustive examples of each theme presented in Tables 3–7. All names are fictitious and bear no resemblance to the children’s actual names.

**TABLE 3:** Described changes in children.

Main theme and subthemes	Short description and examples
**Summary**	**Eight families described positive changes in their child after therapy, including changes in agency, exploratory behavior, improved regulation and changes in affective range and expressivity**

**Main theme: Perceived changes in behaviors related to self-agency and exploration**	
***Increased self-agency, exploration, and daring***	**Four families described their children as taking more charge of events, being more daring, or expressing more exploratory curiosity** *I: But what - can you say something about that- what has improved, the changes and - how do you see it?* *Anne’s mother: Hm… She can go on an airplane* *I: Yes* (*laughs a little*). *Yes, that is VERY important* *Anne’s mother: She can… stay overnight with grandparents without us being there…*
	*Kathryn’s mother: And - but then we sat on the plane home now, and then she says to me: Mom, you know what - we’re going on - we’re going to the mountains - We’re going to the mountains for camp. And she has not said that before. She has always - if she has said it, then it has been like – oohh* [*anxious-sound*] *- or* [*she’d*] *rather not talk about it. But now she told me she was going to camp –*
	*John’s mother: …because we have seen a change. Even though we still - he can still have some anxiety attacks* *I: Yes* *John’s mother: As we - as we see. But there has been a change, and he is perceived as more solution-focused.*
**Main theme: Perceived changes in the regulation of arousal and behavior**	
***Improved regulation of arousal and behavior***	**Six families described signs of improved regulation of arousal states and behaviors** *Sean’s mother: Yes. Yes, he sort of manages to calm down faster now, I feel. Calms himself a bit*.
	*John’s mother: The first change we saw was that he started eating a little more.*
	*Anne’s mother: And then… She can still slam doors and quarrel over the most insignificant things* *I: Yes* *Anne’s mother: With both siblings and parents* *I: I do not think we can -* (*laughs*) *Anne’s mother: No. But it kind of blows over* *I: Yes* *Anne’s mother: It does not last a day –*
	*Vicky’s mother: It has been a lot - she is not SO… that tension in the body, in a way* *I: Yes* *Vicky’s mother: It - she’s calmer.*
***Less aggression and outbursts***	**The improved regulation could be seen through less aggressive behaviors or outbursts** *I: So there have been fewer outbursts?* *Vicky’s mother: Yes* (…) *Vicky’s mother: And less… like before, she could be very, like, when she had her outbursts that she could approach us physically, I was going to say. That - not that she attacked me* *I: No* *Vicky’s mother: Simply that she came so close that she walked into me or was a bit like that - And she has not done that at all anymore.*
	*Ella’s mother: And [*previously*] she would go into her brother’s room to tear down his shelves when they fought or quarreled* *I: Yes* *Ella’s mother: Typical sibling quarrel* *I: Yes* *Ella’s mother: “Now I go and tear them down,” And then she runs in there, and then I just wait for it – the crash, right* *I: Yes* *Ella’s mother: And it is not so long ago. And - but she did not tear down the shelf*
**Main theme: Perceived affective and reflexive changes**	
***Perceived changes related to affective range and expressivity***	**Eight families described positive changes in the child’s affects** **This included families describing an extended range of emotions in the child** *Anne’s mother: While now I think maybe she just has so much more* *I: Yes, or that maybe it’s not just anger* *Anne’s mother: Right. There may be other feelings as well* *I: Yes, it’s very easy to get angry* – *Anne’s mother: But now she has started talking about the other emotions as well, which are not just anger.*
	*Ella’s mother: She expresses… more emotions now than before* *I: Yes* *M: It’s not just anger –*
	*Sean’s mother: - and he has become much better at talking* *I: Yes*
	*Sean’s mother: - and put into words what is upsetting to him.*
***Improved expressivity and regulation of particularly negative affect***	**Particularly communication and regulation of anger and anxiety were described as improved** *Ella’s father: Because she says a lot more - she says when she’s angry or upset or* *I: Yes* *Ella’s father: Because before* [*therapy*] *it was just screaming and breaking things*
	*John’s mother: Because before I had the impression that the anxiety just took him “whooooosh“* *I: Yes* *John’s mother: While NOW he can talk about it* [*the anxiety*]. *You see that it* [*anxiety*] *builds up, yes* *John’s father: Yes* *John’s mother: But he can talk a little more about it along the way now*
***Increased reflexivity around internal states***	**Three families described children seemingly reflecting more on internal states in themselves and others** *John’s mother: No, I think - for the boy, I think it has been a process of raising awareness in one way or another* *I: Yes* *John’s mother: To become aware that he has experienced things that have been tough. And also maybe a small awareness that it is possible to talk about it.*
	*Anne’s mother: And then the girl has managed to use HER* [*therapy*] *focus to correct me* *I: Yes* (*Both laugh a little*) *Anne’s mother: And she’s right in that I – for an adult… I guess I could also benefit from regulating my* [*blurry*] *a little better*
**Main theme: Thoughts about the change**	
***Change linked to therapy***	**The changes were seen as linked to therapy and greater contact with inner experiences** *Ella’s mother: I experience that she can express emotions in other ways than just anger* (…). *It* [*therapy*] *made her more familiar with herself and more in contact with what was inside herself.*
	*John’s mother: Yes, so it* [*therapy*]*… it is a process of becoming aware…* *I: But do you think he thinks that THAT is what he has done here? Therefore* *John’s mother: Yes* *I: - received help to* *John’s father: Yes, I think so*
***Uncertainty about change***	**Three families wondered about change as a factor of time or were unsure about the longevity** *Anne’s mother: It may well be that she - I think she would have had some improvement without going here as well because part of it was related to my illness and such* *I: Yes* *Anne’s mother: And just the fact that time goes by helps* *I: Yes* *Anne’s mother: But - but I think it has helped to go here too –*
	*Ella’s mother: Yes. But then I think - I know that… - how long does it last, sort of? Has it had a lasting effect on her, or –*

*I, interviewer; all names are fictitious.*

**TABLE 4:** Described changes in parents.

Main theme and subthemes	Short description and examples
**Summary**	**Parents from eight families described changes in themselves or their parenting, including being more sensitive or patient as a parent. Parents from five families described being able to see their child and their needs more clearly**
**Main theme: Perceived changes in parents**	
***Perceived changes in sensitivity and parenting***	**Parents from eight families described changes in themselves or their parenting, including being more sensitive, patient, curious, or developmentally supportive as a parent** *Monica’s father: For MY part, I became a little aware that maybe I also have to make some changes in relation to yelling at her and - not rise up* [*in anger*] *right away, but count to 10…*
	*Sean’s mother: But… then I have become a little better as well, to try to… bring out the positive and…*
	*I: No. So you have become more aware of YOUR function?* *Siri’s mother: Yes, I think so* *I: Yes* *Siri’s mother: I think we have become more… more - yes - I think so - On things that are not about school, and a little in connection with school… so we practice – in a way to applaud the process and not the results*
	*Anne’s mother: …not least we parents have become a little more aware of - yes, a little more consistent in what we do and - yes - how we put [*things*]…*
***Seeing the child clearer***	**Parents from five families described being able to see their child differently or more clearly. This included becoming more aware of their children’s vulnerabilities and needs. This was also linked to changes in the child’s affective expressivity** *John’s mother: I got a kind of eye-opener that things were perhaps a little more serious than we thought.*
	*Siri’s mother:* [*she is*] *Very dutiful, very - very… Maybe MORE dutiful than we really understood.*
	*Ella’s mother: I think it’s easier to understand her - to see her now* *I: Yes* *Ella’s mother: But it’s… also because she can more easily express herself*

*I, interviewer; all names are fictitious.*

**TABLE 5:** Described changes in transactions.

Main theme and subthemes	Short description and examples
**Summary**	N**ine families described positive changes in transactions with their children including less quarreling and more dialogue. The changes were attributed to experienced changes in the understanding and perception of the child and more awareness around parenting strategies, changes in the child’s affective expressivity and changes in the parents’ understanding of their own part in difficult transactions**

**Main theme: Perceived changes in transactions between child and parents and presumed contributors**	

***Increased understanding of the child and helpful strategies contributing to more positive transactions***	**Parents from eight families reported an increased understanding of the child and helpful strategies to meet the child that made transactions more positive** *Ella’s mother: And not least that WE also got to know her better* *I: Yes* *Ella’s mother: And learned to meet her in other ways* *I: Yes* *Ella’s mother: And the communication got better because she tells me too* *I: Yes* *Ella’s mother: - that now I’m angry, or now - you do not listen to me!*
	*Matt’s father: He’s still anxious, and he is - he’s a VULNERABLE boy. But we have learned a little how to talk to him about it*

***Changes in perceptions contributing to improved dialogue***	**Parents from five families described how changes in perceptions of the child contributed to improved dialogue and more relaxed children** *Kathryn’s mother: And the girl also knows - we talked about it - that she feels that we as parents meet her in a different way* *I: Yes. Right* *Kathryn’s mother: And that makes her relax more. She had to - like this with us looking at the girl as a problem, because we have done that. She had become a problem for us in some contexts* *I: Yes* *Kathryn’s mother: But that we now just have to set - set aside that she is a problem, and lower the bar in relation to our demands… So that it is about US* *I: Yes* *Kathryn’s mother: And really, we should have understood THAT too* (*laughs a little*) *- that it’s not just about the girl getting some tools – “here fix this. You are 11 years old” - it does not work.*
	*Matt’s father: And* [*we have learned*] *that he must be allowed to talk about - that he has his drawing, which is an outlet for this unrest he has inside* *I: So there has been a change in - with you* *Matt’s father: Yes* *I: - in how can you both help him, meet him, understand him too?* *Matt’s father: Yes, yes* (…) *Matt’s father: And he is more relaxed now. He is not trying to hide it* [*his drawings*] *anymore* *I: Yes* *Matt’s father: So it’s like HE should not feel bad for expressing himself as he does* *I: Yes, exactly* *Matt’s father: It’s a cry for something* *I: Yes. Yes, like that - that’s how you think, yes* *Matt’s father: Yes* *I: Yes* *Matt’s father: And then maybe we can talk to him a little - why did you draw so much blood today?*

***Changes in the child’s affective expressivity contributing to improved dialogue***	**Parents from five families described how changes in the child’s expressivity and sharing of emotions contributed to improved dialogue and less conflict** *Vicky’s mother: So it is - there have been fewer of those big outbursts. Of course, it still happens, but* *I: Because it was some of her - I do not remember them apart* *Vicky’s mother: Yes. Yes, because she’s not saying much and not saying much, and then all of a sudden, everything just exploded* *I: Oh yes. OK* *Vicky’s mother: Yes. So I think it has become smoother, that in a way we talk more along the way, and it is not so much where the whole world is wrong, and there is a full crisis when something happens. So it - yes* –
	*Anne’s mother: Yes, she’s gotten better at coming and talking about it to dad or me* *I: Yes* *Anne’s mother: Before* [*therapy*], *she could just suddenly become incredibly upset* *I: Yes* *Anne’s mother: - and say ugly things* *I: We know* *Anne’s mother: - without us realizing what it was, and then we found out 2 days later what had happened*
***Changes in the parents understanding of their role contributing to less conflict***	**Parents from six families described how changes in the parents’ understanding of their own part to play in difficult transactions were seen as important contributors to changes in the child’s behaviors** *Monica’s father: That - that I have become more aware of leaving my work voice at work and* *I: Yes* *Monica’s father: - rather be a little softer at home* *I: But have you seen that it has worked? ONE thing is that you think it works* *Monica’s father: We don’t quarrel as much.*
	*Siri’s mother:* (…) *So sometimes we say things and do things and - and focus on things that we do not think are performance-focused, but that will be that for her* *I: Yes* *Siri’s mother: So, for example, cleaning her room or… or running in with shoes on - that is, things like that -… Things that you can comment on that she does not need to get a comment on* *I: Yes* *Siri’s mother: At least not in a negative sense*

*I, interviewer; all names are fictitious.*

**TABLE 6:** Factors parents described as helpful or not.

Main themes and subthemes	Short description and examples
**Summary**	**Parents mentioned both child and parental sessions as important. They also mentioned play and creativity and the therapy-focus. No factors were described as unhelpful, but attendance could be challenging, and play needed to be adapted to the individual child**

**Main theme: Parallel parent and child sessions**	

***Parallel parent and child sessions were important***	**Parallel parent sessions were mentioned as important by seven families. Here gaining new understanding and new tools and insights through reflection was emphasized** *Kathryn’s mother: We were taken care of, and then slowly but surely, we began to reflect in a way on… how to do it, etc. –* [*blurry*] *on the situation* *I: Yes* *Kathryn’s mother: - more than when she came* [*to therapy*].
	*Siri’s mother: I think - Yes, I think so - maybe what has been most important, it is that the dad and I have talked about this and thought about what it is WE do, what it is WE in a way expose her to?*
	*Ella’s mother: I think it is very positive that we as parents were also offered sessions*

***Parent sessions need tailoring to different needs***	**Attending parent sessions could pose challenges and the number of sessions needed tailoring to individual families** *Kathryn’s mother: …it has been very tough at times, in that I teach* *I: Yes* *Kathryn’s mother: - and my workplace operates at a financial deficit, and you do not feel like charging it with substitute hours and* *I: No* *Kathryn’s mother: So that’s a thing - a dilemma, that we would like to have it after school-hours, for MY part at least.*
	*I: Do you think it was too FEW sessions? Could you have wanted more? …* *Vicky’s mother: I do not know* *I: No* *Vicky’s mother: Maybe, sometimes, in a way, but… No, my husband is glad that there were no more, in a way, HE is the one who has been at work while I have driven the girl. So for HIM, it has been in a way very good that there have not been more. I may have thought… that it would have been nice to get a little more input along the way* *I: Yes* *Vicky’s mother: Yes. So a bit of both. Yes*

***Intersubjective and therapeutic potential of parent sessions***	**The collaborations with the therapists were described as supportive and positive overall. Parent sessions were also described as having therapeutic potential by one family**

	*Kathryn’s father: It was actually - because we thought there would be a lot of focus on the girl, but… you notice yourself too - I have never experienced anything like that - but so… it is kind of therapeutic for oneself too* *I: Yes* *Kathryn’s father: to be an hour - because you examine yourself* *I: Yes, yes, of course* *Kathryn’s father: Because - for me - especially since I have had a bit of this here with… when I was little - a bit similar* *I: Yes* *Kathryn’s father: - maybe not in that - NOT to that extent, but - but also a bit of anxiety, etc. - so to delve into the depths of oneself* *I: Yes* *Kathryn’s father: - it has actually done quite good.*
	*Monica’s father: So I feel that* *Monica’s mother: It has been a good collaboration* *Monica’s father: Very! I feel it* *I: Yes* *Monica’s father: I feel that if we had something… I think we would have been met. That’s the feeling I have*

**Main theme: Play and creativity**	

***Play and creativity as positive***	**Play and creative activity were explicitly mentioned as positive by four families** *Vicky’s mother: Because - you play there a little. That’s probably it… play with some meaning* *I: Yes* *Vicky’s mother: The thing about being….* [*they*] *talk a little indirectly about the feelings by doing some symbolic things - like the volcano in the sandbox and such*.
	*Matt’s father: So we think this play approach is very ingenious*
***Play as a source of information***	**The content of the play or creative activity could function as a gateway to gaining a better understanding of the child**
	*Matt’s father: And then the boy drew a drawing for* [*the therapist*] *and took it with him, and he* [*the therapist*] *kept it, so there was a conversation about it* [*the drawing*] *I: And it was with blood, right?* *Matt’s father: Yes. There was blood and war and shooting and stuff* *I: Yes* *Matt’s father: So - and that was when we concluded that this is an outlet for the boy to express what he has inside* *I: Yes* *Matt’s father: It’s a way to get it out*

***Play needs tailoring***	**At the same time, one family described that play wasn’t necessarily right**
	*Siri’s mother: But we probably mentioned she had* [*previously*] *been here in connection with the fact that she has had a phobia* (…) *Siri’s mother: And they have played with moon-sand* [*in therapy*], *and she thought it was great fun* *I: Yes* *Siri’s mother: But she does not quite see what it’s going to do* (…) *Siri’s mother: So that she probably felt that she was really working on something that was important to her* *I: Yes, that time* *Siri’s mother: - when she was exposed to the thing she was afraid of* [*in previous therapy*] *I: Yes* *Siri’s mother: While she does not understand what she is doing this* [*play in therapy*] *for*

**Main theme: Therapeutic focus**	

***Child-centered therapy-focus seen as positive***	**Three families mentioned the explicit tailoring to the child through the therapeutic focus as positive** *Vicky’s mother: It became so concrete, so it was so easy for her to relate to what it* [*the focus for therapy*] *was, and… Yes –*
	*I: Yes, the focus - when we talk about it - was that something you - I was about to say found useful? Did it seem to* *Anne’s mother: Yes, yes, useful. Very - very concrete and straightforward, and it can* *I: Yes* *Anne’s mother: And not least* [*increase the*] *understanding for both adults and children –*

*I, interviewer; all names are fictious.*

**TABLE 7:** Other salient themes.

Other salient themes:	
**Summary**	**Two themes stood out as salient and relevant. This was a view of therapy as the start of a continuing process, and a desire for follow-up**
**Main theme: Seeing therapy and change as a developmental process**	
***Seeing therapy as the start of a process***	**Three families described therapy as the start of a process** *Kathryn’s father: Yes, but - well… you think like that - you have to find a SOLUTION - when you finish you find a solution* *I: Yes* *Kathryn’s father: But it’s not THAT easy* *I: No* (*laughs a little*) *Kathryn’s father: It really isn’t…* *I: No* *Kathryn’s father: No. But feel we have got a - something to grasp on to, that we have actually gotten a little to the depth* [*of the problem*] *and – and can approach things differently*.
	*Matt’s father: Because this is not some magic trick that you can do, and then he becomes very confident and all that* *I: No* *Matt’s father: It’s a process that’s starting* *I: Yes* *Matt’s father: So, we just have to continue*
***Increased patience and understanding for the child’s developmental process***	**Viewing therapy as a process could increase patience and reinforce a developmental perspective** *Kathryn’s mother: It’s a bit like he says, that I think we’ll get there* *Kathryn’s father: Yes* *Kathryn’s mother: We just have to give it time* *I: Yes. So you have - you have not come - you are not all the way there* *Kathryn’s mother: No* *Kathryn’s father: No* *I: But it sounds like you have started something that you think may be..* *Kathryn’s mother: Yes* *Kathryn’s father: Yes* *In: - on a good path then* *Kathryn’s mother: Yes* *Kathryn’s father: Yes* *Kathryn’s mother: So when she is 40, then -No -* [*laughs, indicates making a joke*].
	*Matt’s father: We hoped it would become a little easier for him* *I: Yes*
	*Matt’s father: And it has. So now it’s just up to me and his mother to somehow expand this feeling, so that it becomes even more sensible and mature eventually*
**Main theme: Further needs**	
***Need for follow-up or safety-net***	**Two families described a desire for follow-up. One family asked for more therapy** *Ella’s mother: But if there is something* (…) *that we feel that this becomes too difficult, and…that is, that something should arise* *I: Yes* *Ella’s mother: Of course, I do NOT hope so* *I: No* *Ella’s mother: Is it possible to get any more sessions then, or is it -?*
	*Matt’s father: It would be good if we could have had a little follow-up in a while* *I: Yes* *Matt’s father: To see if it is - because I have SO little desire for him to fall out of the system again* *I: Yes, I understand* *Matt’s father: Because it takes so long to get him back in*.
	*John’s mother: So, we hope that we will still have some offer* [*of therapy*] *in the fall*

*I, interviewer; all names are fictious.*

### Parents Expectations Regarding Therapy

#### Main Theme: Expecting Help

*Subthemes: Giving the child a space to talk; Giving the child tools for regulation; Helping the parents see, meet, and regulate their child better.*

An analysis of parental expectations before the start of therapy showed that the parents did not have very detailed expectations regarding therapy. All the families expected to get some form of help, even if it did not fix everything. In terms of the particulars of the help they expected, eight of the nine families expected their child to receive a safe space to talk and reflect about their thoughts and feelings. Four parents talked about how they expected therapy to give the child new tools for self-regulation, including an expanded emotional vocabulary to avoid outbursts, or ways the children could calm their physiology when anxious or agitated. Four families said they expected practical supervision on how to better meet or regulate their child themselves.

### What Changes, If Any, Did Parents Describe in Their Children After Time-Limited Intersubjective Child Psychotherapy?

Eight families described positive changes in their child after therapy, including changes in behavior, such as increased agency, exploratory behavior, and regulation. The themes, descriptions, and examples are presented below and further in Table 3.

#### Main Theme: Perceived Changes in Behaviors Related to Self-Agency and Exploration

*Subtheme: Increased self-agency, exploration, and daring.*

Parents from four families described their children as taking more charge of events, being more daring, or expressing more exploratory curiosity, like aiming to have a school-vaccination unaccompanied by a parent, spending a night outside of the home or going to camp, or trying to take part in a school performance despite anxiety.

#### Main Theme: Perceived Changes in the Regulation of Arousal and Behavior

*Subthemes: Improved regulation of arousal and behavior; Less aggression and outbursts.*

Six families described improved regulation of arousal as evident in improved sleep and appetite, less aggression, or less frequent outbursts of shorter duration or less intensity.

#### Main Theme: Perceived Affective and Reflexive Changes

*Subthemes: Perceived changes related to affective range and expressivity; Improved expressivity and regulation of particularly negative affect; Increased reflexivity around internal states.*

Eight families described a seemingly wider range of affects in the children, and better affect expressivity and reflexivity, particularly for anger and anxiety. Three families described children seemingly reflecting more on internal states in themselves and others, for instance, by using things they learned in therapy to regulate their parents or talking more about mental states in addition to affects.

#### Main Theme: Thoughts About the Change

*Subthemes: Change linked to therapy; Uncertainty about change.*

Two families explicitly stated that they saw the changes as linked to increased contact with and awareness of inner experiences after therapy. Three families had some uncertainty about the changes, thinking they might be at least partially a factor of time, or they were unsure about the expected longevity of the changes. One family was unsure of the extent of changes in the child and described more positive changes in the parents than in the child. The ninth family only described positive changes in the parents, but said the parental change was the most important outcome for them.

### What Changes, If Any, Did Parents Describe in Themselves After Time-Limited Intersubjective Child Psychotherapy?

The main themes, descriptions, and examples are presented below and further in Table 4.

#### Main Theme: Perceived Changes in Parents

*Subthemes: Perceived changes in sensitivity and parenting; Seeing the child clearer.*

Parents from eight families described changes in themselves or their parenting, including being more sensitive, patient, or consistent as parents. The changes included being less angry, focusing more on positive affect, or applauding the child’s attempts rather than results. Parents from five families described being able to see their child differently or more clearly. The parents said that changes in the child’s affective expressivity made it easier to see the child, but the parents also reported becoming more aware, curious, and reflexive around their children’s vulnerabilities and needs.

### What Changes, If Any, Did Parents Describe in Transactions Between Themselves and Their Child After Time-Limited Intersubjective Child Psychotherapy?

The main themes, descriptions, and examples are presented below and further in Table 5.

#### Main Theme: Perceived Changes in Transactions Between Child and Parents and Presumed Contributors

*Subthemes: Increased understanding of the child and helpful strategies contributing to more positive transactions; Changes in perceptions contributing to improved dialogue; Changes in the child’s affective expressivity contributing to improved dialogue; Changes in the parents understanding of their role contributing to less conflict.*

The parents from all nine families described positive changes in their transactions with their children, such as less quarreling and more dialogue. These changes were attributed to an increased understanding of the child and a better understanding of parenting strategies for their child’s individual needs, such as recognizing that the child needed lower demands or more support. Five families expressed how positive changes in their perception of the child made their children lower their guard or improve the transactions. For instance, one family stopped seeing their child as a problem noting how this changed their behavior and relaxed the girl. Another family stopped seeing their child’s expressions as frightening, which meant the child could stop hiding their expressivity, opening possibilities for dialogue. Information from child sessions and parent sessions (and their coherence) contributed to better understanding the child, as did changes in the child’s affective expressivity. Finally, six families reported that changes in the parents’ understanding of their part in challenging transactions contributed to less negative transactions.

### What Did Parents Describe as Helpful or Not in Time-Limited Intersubjective Child Psychotherapy?

The main themes, descriptions, and examples are presented below and further in Table 6.

#### Main Theme: Parallel Parent and Child Sessions

*Subthemes: Parallel parent and child sessions were important; Parent sessions need tailoring to different needs; Intersubjective and therapeutic potential of parent sessions.*

Parallel parent sessions were mentioned as important by seven families. Here, gaining new understanding and new tools and insights through reflection and the timely and thematic coherence with the child sessions were emphasized. The collaborations with the therapists were described as supportive and positive overall and with possible therapeutic potential. No factors were mentioned as unhelpful but attending parent sessions could be practically challenging for some families. Some families wanted more parent sessions or a different spacing of sessions, some felt it was the right amount, and some wanted fewer parent sessions.

#### Main Theme: Play and Creativity

*Subthemes: Play and creativity as positive; Play as a source of information; Play needs tailoring.*

Play and creative activity were explicitly mentioned as positive by four families. They described play and creative activities in therapy as allowing the children to explore and express their feelings with or without words. They also described how the play’s content could function as a gateway to better understanding the child. At the same time, one family described that play was not necessarily experienced as positive or meaningful for their child who struggled with a phobia, indicating a problem with working alliance due to the perceived irrelevance of the therapeutic tasks.

#### Main Theme: Therapeutic Focus

*Subtheme: Child-centered therapy-focus seen as positive.*

The therapeutic focus was mentioned as helpful by three families. The focus was described as helping increase the understanding of the therapeutic work for both parents and the child. The focus was also described as highly individually adapted, helping the child relate to and engage with the therapy.

### Did the Parents Mention Other Salient Themes of Relevance?

Two additional themes stood out as salient and relevant in the interviews. The main themes, descriptions, and examples are presented below and in Table 7.

#### Main Theme: Seeing Therapy and Change as a Developmental Process

*Subthemes: Seeing therapy as the start of a process; Increased patience and understanding for the child’s developmental process.*

Three families described therapy and change as a process with no clear endpoint or magic fix. This view was accompanied by increased patience and a more developmental focus on the child’s difficulties, including the parent’s role in helping to expand the child’s experiences further.

#### Main Theme: Further Needs

*Subtheme: Need for follow-up or safety-net.*

Two families described a desire for follow-up or a safety net in terms of an open door if the child showed increased difficulties. One family asked for more therapy.

## Discussion

Child psychotherapy has certain characteristics that differ from adult psychotherapy, including the importance of including caregivers in the process (Slade, 2008; Hafstad and Øvreeide, 2011). In addition, psychotherapy with children needs to consider developmental and transactional processes both during therapy and when assessing treatment efficacy, instead of only focusing on symptom relief (Jacobsen and Svendsen, 2010; Johns and Svendsen, 2016). Therefore, the current study investigated parental perceptions of change after TIC through nine qualitative interviews with 13 parents of nine children that had received TIC for internalizing difficulties. An investigation of the families’ expectations before therapy showed that all the families had positive expectations regarding therapy. Almost all the families (eight) described expecting their child to be given a space to talk about their feelings and thoughts. About half expected the child to receive tools to self-regulate better, and as many expected to receive tools or supervision that could help them become better able to see, meet or regulate their child as parents.

In the analysis of the interviews after therapy we focused on the parents’ perception of changes in the child, in the parents themselves, and their transactions after TIC. We also looked for parents’ descriptions of helpful aspects of therapy and other salient themes in the interviews. The discussion will focus on each of the five research questions in turn before ending with an overall reflection on how different parts of therapy, and their synergy, could help facilitate the changes. The research questions were: (*1*) *What changes, if any, did parents describe in their children after TIC?* (*2*) *What changes, if any, did parents describe in themselves after TIC?* (*3*) *What changes, if any, did parents describe in transactions after TIC?* (*4*) *What did parents describe as helpful or not?* (*5*) *Did parents describe other salient themes of relevance?*

### Parents’ Description of Change in Their Children After Time-Limited Intersubjective Child Psychotherapy: Increased Agency, Regulation, Affect Integration, and Reflexivity

The families in the current study described positive changes in all but one child after TIC. In the last family, changes in the parents and their ability to support the child were deemed as the most important outcome. Three families had some uncertainty about the changes, thinking it might be partially a factor of time, or they were unsure about the longevity of the change. Although families were asked to report any signs of change, none of the families described negative changes.

Overall, the changes described by the families were consistent with changes in processes important for healthy development. For instance, several parents described their children as taking more agency in shaping events, some described an increase in positive curiosity around future events previously associated with anxiety, and some described their children as more daring. The described increases in the children’s ability and interest in challenges indicate an expansion toward higher self-agency and exploration. This expansion could provide developmental benefit after therapy is terminated as well, as self-agency and exploration are key factors in healthy development (Stern, 1985/2000).

In addition, several families described their children as better able to regulate arousal and affect, core developmental processes often negatively affected in children seeking therapy (Jacobsen and Svendsen, 2010) and involved in a large proportion of mental disorders (Jazaieri et al., 2013). The parents described the children as calmer, less anxious, more harmonious, and more secure after therapy. They spoke of children that were faster to calm down with fewer or less aggressive outbursts. Such changes can make life easier for children and their families, leaving more room for dialogue and healthy development. Some families reported changes in functions affected by regulation in the autonomic nervous system, such as appetite, weight, and sleep, implying positive improvements in autonomic regulation as well. As autonomic regulation can significantly impact mental and physical health over time (Beauchaine and Thayer, 2015; Kemp et al., 2017), these changes are important for development. In particular, helping children move away from unpleasant states of autonomic dysregulation (such as fight-flight responses) is vital for the ability to engage and connect relationally (Porges, 2015). In addition, improvements in autonomic regulation may reduce long-term negative impacts on physical health stemming from psychological dysregulation (Kemp et al., 2017).

Affect is another important part of development that is often disrupted or disturbed in psychopathology. Affects help magnify and clarify the significance of internal and external events and are a crucial source of information, motivation, and communication (Saarni et al., 1998; Jacobsen and Svendsen, 2010). Most of the parents described wide-ranging affective changes in their children after TIC, all positive. The children were described as seemingly aware of a wider range of affective states. In addition, they were described as better able to tolerate and regulate their emotions. The children were also described as having found more adaptive ways of expressing and communicating how they felt. In particular, the children were perceived as expressing previously overwhelming emotions, such as anger and anxiety, more clearly and with less aggression or intensity after therapy. The described changes included changes to the children’s verbal and behavioral expressivity, such as expressing their feelings more clearly and using words instead of physical aggression. The described affective changes indicate that the parents saw signs of higher affect integration (Solbakken et al., 2011b) in the children after therapy. Higher affect integration includes higher awareness, tolerance, regulation, and verbal and nonverbal expressivity of affect, allowing for more precise communication of emotional and developmental needs. Higher affect integration contributes to supportive interactions within interpersonal relationships and higher intra-personal clarity in reference to internal and external events and is highly important for development (Monsen and Monsen, 1999; Solbakken et al., 2011a). The current results mirror earlier results (Johns, 2018; Fiskum et al., in review^1^) showing higher affect integration after therapy when interviewing children from the main study with the affect consciousness interview (Monsen et al., 2013; Taarvig et al., 2015). That the described changes in this study were particularly salient for anger and anxiety is also consistent with earlier results showing that integration of these affects can be especially challenging in internalizing disorders (Taarvig et al., 2016; Fiskum et al., 2021). Finally, the processes involved in affect integration are important for and related to reflexivity and mentalizing (Fonagy et al., 2002; Solbakken et al., 2011b). In line with this, several of the children were described as more able to reflect on internal states in themselves and others, making them more relationally competent.

### Parents Description of Changes in Themselves After Time-Limited Intersubjective Child Psychotherapy: Increased Sensitivity, Reflexivity, Intersubjective Capacity, and Patience

Most of the parents described changes in the perception of themselves and their parenting after TIC. The main perceived change was a sense of being better able to see and understand their child. This change was tied to both the child’s increased affective expressivity and increases in the parents’ curiosity and awareness of their children’s vulnerability and developmental needs. Several parents described seeing their child’s challenges more clearly, with two parents realizing things were more difficult than previously thought. Other parents described becoming more aware of their children’s tendency toward being overly conscientious or needing more physical affection than previously believed. Furthermore, several parents described themselves as more reflexive around their parental practices and better able to provide more sensitive parenting, such as meeting their children on their specific needs. Several parents described how changes in how they perceived their child led to important changes in parental behaviors and thoughts, such as lower demands, less yelling, and more sensitive or affectionate parenting. For instance, one father described the main effect of therapy as an understanding that his interactions with the child had been overly negative and that he had to change his communication to reduce the child’s challenges. In addition, one parent mentioned that the parent sessions could be therapeutic for the parents as well. In this case, the parent found the sessions helpful in examining his own experiences with feelings of anxiety. Several other parents mentioned feeling seen and met and that the sessions were a positive experience. These descriptions suggest that parent sessions may provide other positive effects for parents, such as strengthened affect integration and experiences of feeling intersubjectively and relationally met. Parents can then bring these experiences into their relationship with their child going forward (Hansen, 2012; Johns and Svendsen, 2016). Finally, several parents described increased patience with the child’s processes of healing and development. This increased patience coincided with an understanding that therapy might not fix everything now but should instead be seen as the start of a developmental process that could take time and continue after the termination of therapy, consistent with transactional models of change (Sameroff, 2009). Such an understanding of change as a gradual process can help parents sustain patience and motivation for the long haul and help the family and the child over time.

Importantly, all the parental changes described are believed to be vital for development. A major goal of psychotherapy with children is strengthening the parent’s ability to see and meet the child’s needs (Slade, 2008; Hafstad and Øvreeide, 2011), thus supporting the development of regulating and reflexive/mentalizing abilities (Fonagy et al., 2002; Schore and Schore, 2008; Midgley et al., 2017). The parents in this study described precisely this; they had become more aware of their child’s feelings and needs, as well as more curious and aware of possible factors behind the child’s behavior, of themselves as parents, and how to meet and navigate the child’s feelings and needs in a more sensitive way. These changes can, in turn, contribute to parenting that increases the child’s own regulating and reflexive capacity (Siegel, 2001; Schore and Schore, 2008; Slade, 2008).

### Parents Descriptions of Changes in Transactions After Time-Limited Intersubjective Child Psychotherapy: Increased Intersubjectivity and Scaffolding and New Opportunities for Development

All nine families described positive changes in the transactions with their children after therapy. Overall, the parents described being more available for, attuned to, and better able to regulate and scaffold their children after therapy, meaning they could support development to a larger degree. They also described positive changes in the children attributed to the transactional changes, including children that were more relaxed and easier to regulate or more open. The described changes in transactions in all the families is, perhaps, the most notable result of this study. A lack of reliable, safe, nurturing, and attuned relationships for co-regulation in childhood can cause severe limitations to a child’s capacity for later self-regulation (van der Kolk and Fisler, 1994; McCrory et al., 2010; Ardizzi et al., 2013; De Bellis and Zisk, 2014), resulting in neurodevelopmental adaptations and psychopathology (Teicher et al., 2021). In addition, negative transactions preceding therapy may persist during and after therapy unless addressed and corrected, serving as maintaining factors for further difficulties. Therefore, psychotherapy with children must aim to address and, when needed, improve important transactions around the child. Our results show that TIC can support development by minimizing negative transactional effects between parents and children. The parents described less negative transactions marked by less conflict, more dialogue, and clearer roles after therapy. Parents also described lowering demands and giving the children more time and space to feel what they felt without forcing change. The transactional changes the parents described imply strengthened intersubjectivity and increased scaffolding in transactions between the parents and their children.

Intersubjective exchanges and transactions, where the individual minds of child and caregiver can dyadically expand (Tronick et al., 1998; Tronick, 2004), help the child organize and regulate in new ways, including internalization of new patterns of activation and updated working models of the world. Being cast in new roles (e.g., going from “a problem” to “a child needing adult support”) also allows for new experiences that further expand the developmental possibilities available for a child and its caregivers. For instance, one family in the study stopped seeing their child as a problem, instead seeing her as a young child with a desire for change and a need for safety, and as the child and the parents sensed this change, things became less problematic. Things were perhaps not entirely solved, but the parents felt they *would* be solved and could give the girl time to grow at her own pace, with less frustration. Similarly, another family stopped seeing the child’s expressions as frightening and became curious and emphatic, seeking connection instead of control when faced with the child’s expressivity. This shift gave new opportunities for dialogue and emotional intimacy instead of the preceding pattern of restriction and concealment. Both families described how the children responded positively to these changes, exemplifying how positive transactional effects can become drivers of further positive development.

### What Parents Described as Helpful: Parallel Sessions, Play, and Therapeutic Focus

Parallel parent sessions were described as important by most of the families. There was no uniform view on the number of parental sessions needed, with some families wanting more and some fewer sessions, indicating that individual tailoring to different families’ needs is essential. No families were negative to parallel parent sessions outside of a concern that they took time and could necessitate more flexibility for some parents to attend. The parents emphasized the importance of gaining a new understanding of their child and how to meet the child through these sessions. They also emphasized the value of reflecting together, both with the therapist and the other parent and in pace with the child’s sessions. Parents described therapy as a learning experience that expanded their views of the child, themselves, and their transactions. These results mirror Haugvik (2012), who found that parent sessions gave parents a space to clearly see and reflect around their children and themselves. As Haugvik, we believe that our results show that the parent sessions can help parents increase their regulating, reflexive, and mentalizing capabilities, all of which are of utmost importance for a child’s continued development (Fonagy et al., 2002; Schore and Schore, 2008).

In addition to the parent sessions, roughly half the families explicitly mentioned play and creative activity as a form of activity well suited for the children to express themselves. The content of the play or creative activity and the room for reflecting on this with the parent-therapist was described as a gateway to gaining a better understanding of the child’s experiences and expressivity. For instance, several families gained new insight when reflecting on the child’s expressivity in therapy, opening new possibilities for dialogue. On the other hand, one family described that their child had found the play activities irrelevant, possibly interfering with the working alliance and the engagement with therapy. This observation confirms that the therapist must sensitively tailor any activities to the individual child and challenges in the referral (Johns and Svendsen, 2016).

Finally, the formulation of a therapeutic focus is an integral part of TIC, and three families mentioned the therapeutic focus as positive, saying the focus helped therapy become concrete, engaging, and relatable for the child, aiding the therapeutic process. The parents’ descriptions coincide with the explicit aim of the focus, which is to help make therapy understandable, relevant, and engaging, thereby strengthening the working alliance (Johns and Svendsen, 2016). No families mentioned the focus as a negative.

### Other Salient Themes of Relevance: Therapy as a Process and Follow-Up

Two other themes stood out as salient outside of the main research questions. These themes were related to an understanding of therapy as the start of a continuous process and a desire for follow-up or a safety-net after therapy. Three families spoke about therapy as a process they had started that would continue after therapy, expressing hope that things would work out with time. In addition, three families mentioned a desire for further follow-up after therapy, either wanting more therapy (1 family) or the possibility of an open door or a safety-net they could use without “having to jump through too many hoops” if the child showed increased difficulties again. The desire for an open-door could be related to the structure of mental health services for children in Norway, which can be challenging to get into. It could also be related to the uncertainty of not knowing if problems can return or a lack of confidence in going on alone. These results underscore the importance of communicating that therapy is a starting point in a process that will also continue after therapy (i.e., “here, we walk *some* of the way together”) (Johns and Svendsen, 2016). At the same time, it is vital to address parents’ concerns around the process that may erode their confidence and their ability to “walk on alone”. Based on our results, it seems wise to offer parents the option of a follow-up consultation if problems reappear within a certain amount of time to help ease the transition into life after therapy and help support parental confidence.

### Creating Therapeutic Change Through Synergy and Transaction, Not Individual Components

In summary, the descriptions of change in our study indicate that TIC can strengthen children’s core developmental processes related to agency and motivation to explore new behaviors. The results also indicate that TIC can strengthen basic regulatory processes from regulation of arousal to regulation of behavior. Furthermore, the parents’ descriptions of change in their children indicated higher degrees of affect integration, including expanded awareness, tolerance, regulation of affect, and improved affect expressivity in the children after therapy. The parents also described themselves as better able to see and meet their children and more sensitive and reflected in their parenting after therapy. Finally, all the parents described positive changes in their transactions with their children.

Many of the described changes may be related to the children’s therapy sessions. The child-sessions in TIC center closely on the child’s expressivity, interests, and desires in the here and now. In addition, the therapeutic focus can help the child gain overview and ownership of their therapeutic process, further strengthening agency. The use of play in TIC might similarly have played an important part, as play can stimulate and strengthen developmental processes such as attention, regulation, affect integration, and mentalization (Siegel, 1999; Lyons-Ruth, 2006; Booth and Jernberg, 2009). Most importantly, the relational space in the child’s therapy sessions constitutes a vast “potential space” (Winnicott, 1991), offering the child new, corrective experiences of sensitive attunement, intersubjectivity, and relational synchrony. This potential space is centered on the child, with the therapist noticing and responding to micromomentary shifts in the child’s attention, affective expressions, or behavioral impulses, always trying to follow closely along and repairing any ruptures in the connection (Johns, 2018). Together the (often) messy experiences of “hit and miss and repair” provide the child vital experiences of not only being an effective agent that can set and explore an agenda, but also that communication and relationships that break down can be repaired, installing safety to explore and venture beyond the already known (Beeghly and Tronick, 2011).

Likewise, the focus on scaffolding developmental processes in TIC means that the therapist can choose to go where the child cannot, for instance, by attending and attuning to, role-modeling, and naming affects that the child has a hard time noticing, tolerating, regulating, or communicating by themselves or in their relationships outside of therapy. These experiences can strengthen affect integration by pulling together different aspects from sensation to behavior and symbolic representation within a safe relational frame. Similarly, a calm, therapeutic presence may affect nervous system regulation positively (Badenoch, 2018; Dana, 2018), so that changes consistent with a less hyper-aroused autonomic state may stem from the experience of safety and co-regulation in therapy itself.

In much the same way, the changes the parents described in themselves, such as seeing their child and themselves more clearly and being more sensitive parents, may be ascribed to the parent sessions. The parent sessions give parents a supportive, intersubjective, and potential space of their own, with potential therapeutic effects. Here the parents can explore and reflect on their feelings toward the child, their relationship, and their role as parents focused on helping them help their child back onto a healthier developmental track.

Consistent with all these possible contributors to change, parents mentioned parallel child and parental sessions, play, and the therapeutic focus as factors contributing to the changes experienced. All of the mentioned factors are individually associated with positive change in therapy (Bratton et al., 2005; Trowell et al., 2007; Haugvik and Johns, 2008; Drisko et al., 2019). However, it is likely that the changes described in our study stem from the *synergy* of all the different parts present in TIC *together*. The child’s therapy sessions might increase the child’s agency, self-other-regulation, and affect integration, making the child a clearer and more competent transactional partner and agent. The information gained from the child’s therapy sessions may also directly contribute to changes in the parents’ understanding and perception of the child. At the same time as the child becomes more apparent to itself and others, the parental sessions may allow parents to reach higher levels of reflection and sensitivity in their parental practices, which coincide in both time and theme with the child’s changes. By having parent sessions *in parallel* with the child’s sessions and close contact between child and parent therapist, the parent therapist can bring in and translate things from the child’s therapy to the parent sessions, creating a bridge between the child’s symbolic play and expressivity in therapy and something the parents can understand and work with at home. In addition, the therapy focus may shift the family’s focus away from problems, symptom avoidance, and negative transactions (which many families may be stuck in) and toward a focus on resources and collaborative exploration and growth (Haugvik and Johns, 2008; Haugvik, 2012; Johns and Svendsen, 2016). In this way, the therapeutic focus can contribute to a mindset centered on positive processes, growth, and mutual contributions to transactions. Finally, the joint sessions with both child and parents can make the therapeutic process and the changes available for joint reflection, so everyone is on the same track at the same time. Based on the insights from the families in our study, we argue that it is the *emergent sum* of all the different pieces in TIC that results in changes, rather than any one piece alone. How much the individual parts contribute to the whole can likely be variable in different children, families, and contexts. Therefore therapeutic change should be understood as a complex, dynamic, and transactional process where the sum is greater than the parts. In such a view, the art of psychotherapy is to be able to connect with, partake in, oversee, and guide the different processes as they unfold.

### Limitations

It is a limitation that the study had a relatively small sample. A further limitation is that seven families declined to participate. Even if the therapists’ assessments indicated that these families had positive outcomes comparable to the families that participated, we cannot exclude the possibility of differences in satisfaction between the families. It is therefore a possibility that the families that participated were particularly satisfied with or invested in the therapy. It is a further limitation that the interviews were variable in their richness. The parents differed in their ability to describe and reflect, and some parents described changes mostly in one area. All interviews contributed to the analysis, however.

## Conclusion

Time-limited intersubjective child psychotherapy is a developmental and transactional approach to psychotherapy that aims to restart or strengthen core developmental processes and get children and parents back on a healthier developmental track. Specifically, TIC aims to strengthen core developmental processes in the child that are important for intersubjective transactions, such as regulation and affect integration. In addition to child-centered individual therapy with the child, TIC provides parallel parental sessions aimed at helping parents see, reflect around, understand, and meet and transact with their children in more developmentally supportive ways than before. Importantly, TIC is not aimed at only one thing, such as symptom-reduction or increased parental mentalization. Instead, TIC is an integrative approach to therapy that aims to affect developmental and transactional processes in both children and caregivers, in parallel. The parents’ descriptions of change and their reflections on the helpful aspects of therapy indicated that is exactly what happened, giving support to TIC as a developmentally supportive therapy that can help improve transactional processes. The children changed, the parents changed, and perhaps most importantly, their transactions changed, meaning the therapeutic processes could come alive outside of the therapy room.

## Data Availability Statement

The transcripts from the interviews have not been approved for public sharing due to their identifiable nature (qualitative data), but the corresponding author will answer any questions about the data and supply anonymized information without undue reservation.

## Ethics Statement

The studies involving human participants were reviewed and approved by Regional Committees for Medical and Health Research Ethics (division North). Written informed consent to participate in this study was provided by the participants or their legal guardian/next of kin.

## Author Contributions

CF performed the analysis, translated the interviews, wrote the manuscript, and prepared the submission. KJ was the principal investigator. UJ contributed as a therapist in the main project. TA, UJ, and KJ read and discussed the analysis and manuscript and gave critical feedback. All authors contributed to the initial planning and execution of the main study the interviews were conducted as part of and approved the submitted version.

## Conflict of Interest

The authors declare that the research was conducted in the absence of any commercial or financial relationships that could be construed as a potential conflict of interest.

## Publisher’s Note

All claims expressed in this article are solely those of the authors and do not necessarily represent those of their affiliated organizations, or those of the publisher, the editors and the reviewers. Any product that may be evaluated in this article, or claim that may be made by its manufacturer, is not guaranteed or endorsed by the publisher.

## Footnotes

1 Fiskum, C., Andersen, T. G., Johns, U. T., and Jacobsen, K. (in review). Changes in affect integration and internalizing symptoms after time-limited intersubjective child psychotherapy — a pilot study. *Front. Psychol. Psychol. Clin. Settings.*
